# Adiponectin-Transfected Endothelial Progenitor Cells Have Protective Effects After 2-Hour Middle-Cerebral Artery Occlusion in Rats With Type 2 Diabetes Mellitus

**DOI:** 10.3389/fneur.2021.630681

**Published:** 2021-03-03

**Authors:** Meiyao Wang, Yan Li, Renwei Zhang, Shuaimei Zhang, Hongliang Feng, Zhaohong Kong, Nadire Aiziretiaili, Zhengjin Luo, Qi Cai, Yan Hong, Yumin Liu

**Affiliations:** ^1^Department of Neurology, Zhongnan Hospital of Wuhan University, Wuhan, China; ^2^Department of Neurosurgery, Zhongnan Hospital of Wuhan University, Wuhan, China; ^3^Guangdong Mental Health Center, Guangdong Provincial People's Hospital, Guangdong Academy of Medical Sciences, Guangzhou, China; ^4^Department of Neurology, Renmin Hospital of Wuhan University, Wuhan, China; ^5^Department of Pediatrics, Zhongnan Hospital of Wuhan University, Wuhan, China

**Keywords:** type 2 diabetes mellitus, acute ischemic stroke, adiponectin-transfected endothelial progenitor cell, angiogenesis, protective effects

## Abstract

**Objectives:** This present study aimed to examine the effects of adiponectin-transfected endothelial progenitor cells (LV-APN-EPCs) on cerebral ischemia–reperfusion injury in rats with type 2 diabetes mellitus (T2DM) and to explore the underlying mechanisms.

**Methods:** Seventy male Sprague–Dawley rats with T2DM were randomly divided into sham, phosphate-buffered saline (PBS), LV-APN-EPCs, LV-EPCs, and EPCs groups. Transient middle cerebral artery occlusion (MCAO) was induced by the intraluminal suture method. After 1 h of reperfusion, the five interventions were performed by tail-vein injections. The modified neurological severity score (mNSS) was used to assess neurological function before and on days 1, 7, and 14 after MCAO. After 14 days, magnetic resonance imaging scanning, hematoxylin and eosin staining, terminal dUTP nick-end labeling staining, Western blotting analysis, cluster of differentiation (CD) 31 immunofluorescence, and enzyme-linked immunosorbent assay were used to evaluate infarct rate, morphological damage, cell apoptosis, and microvessel density.

**Results:** Compared with PBS, LV-EPCs, and EPCs groups, the LV-APN-EPCs group showed significantly lower mNSS score, lower infarct rate, and less morphological damage (all *P* < 0.05). In addition, compared with other groups, the LV-APN-EPCs group had significantly increased levels of B cell lymphoma/leukemia-2 (Bcl-2) protein, CD31+ microvessels, endothelial nitric oxide synthase, and vascular endothelial growth factor, and decreased levels of Bcl-2-associated X protein and neuronal apoptosis in the peri-infarct cortex (all *P* < 0.05).

**Conclusion:** These results suggest that LV-APN-EPCs exert protective effects against cerebral ischemia–reperfusion injury in T2DM rats by increasing angiogenesis.

## Introduction

Ischemic stroke leads to disability and death worldwide (1), and type 2 diabetes mellitus (T2DM) is one of the prominent risk factors (2). The risk of stroke in patients with T2DM is increased 2-fold compared with individuals without T2DM (3). The comorbidity of T2DM and stroke results in extensive neurovascular damage, impairment of stroke recovery, and stroke recurrence (4, 5). US Food and Drug Administration (FDA) only approved tissue-type plasminogen activator (tPA) as the treatment for acute ischemic stroke. However, intravenous administration of tPA within 3–4.5 h of stroke onset is challenged because of its narrow treatment time window. Above all, diabetes attenuates the effects of tPA on stroke (6). Thus, there is an urgent need to develop ischemic stroke treatments specifically targeting T2DM patients. Cell therapy has emerged as a novel treatment for experimental stroke (7, 8).

Endothelial progenitor cells (EPCs), which were first discovered in 1997 (9), are now recognized as playing a significant role in promoting neurovascular repair and improving long-term neurological function (10). EPCs reside primarily in the bone marrow and can be mobilized into the bloodstream when tissue ischemia occurs. Then, they home and migrate to the region of vascular damage and differentiate into mature endothelium to contribute to neovascularization and reendothelialization. A decreased number of circulating EPCs can predict the poor outcome of vascular disease as the number of circulating EPCs is a biomarker for prediction of vascular outcomes (11). Patients with T2DM have a decreased level of circulating EPCs, which exhibit impaired proliferation and adhesion (12). Therefore, to increase the amount of circulating EPCs and promote their functions in endothelial repair, the transplantation of EPCs is a promising treatment for ischemic stroke in diabetic patients.

Adiponectin (APN) is an adipocyte-secreted adipokine that has received a particular focus because of its insulin-sensitizing and antidiabetes properties (13). Apart from its metabolic functions, APN also exerts some protective effects on the alleviation of stroke (14). APN alleviates stroke primarily through activating endothelial nitric oxide synthase (eNOS) (15). The evidence also demonstrates that APN may participate in regulating the functions of EPCs, which include proliferation, migration, and cell differentiation (16, 17). In our previous study, we have reported that gene-modified cell therapy may be a useful approach for the treatment of ischemic stroke in non-diabetic rats (18).

In the present study, lentivirus constructs expressing the green fluorescent protein (GFP) and APN gene directed against EPCs (LV-APN-EPCs) were transplanted into T2DM rats after the so-called middle cerebral artery occlusion (MCAO) to evaluate the therapeutic efficiency and underlying mechanism of LV-APN-EPCs treatment in T2DM rats after the ischemic stroke.

## Materials and Methods

All experimental procedures followed the *Guide for the Care and Use of Laboratory Animals* published by National Academies Press (US) (8th edition, 2011). The use of animals was approved by the Animal Ethics Committee of the Medical School of Wuhan University. Investigators who conducted the experiments, collected data, and assessed outcomes were blinded to the treatment allocation throughout the experiments.

### Animals

Seventy male Sprague–Dawley (SD) rats aged 5 weeks and weighing 150–160 g were purchased from Hubei Research Center of Laboratory Animals (SCXK [E] 2015-0018). The rats were housed in a certified animal care facility, and housing conditions (5 animals per cage, 22 ± 2°C, 55 ± 5% relative humidity, 12 h artificial light/dark cycle) with *ad libitum* access to water and regular Rat chow (Huafukang, Beijing, CN) for 1 week to adapt to the laboratory environment. Food intake and body weight were measured on a weekly basis.

### T2DM Induction

T2DM was induced using a combination of 2-week high-fat diet (Research Diet D12492 60 kcal% fat, USA), followed by a low dose (35 mg/kg) of streptozotocin (STZ, Sigma Chemical Co., St. Louis, MO) intraperitoneal injection and continued a high-fat diet for another 2 weeks (19). The fasting blood glucose was measured before STZ injection and 2 weeks after STZ injection by using a glucometer (Johnson & Johnson Co., New Brunswick, NJ, USA). The T2DM rat model was successfully established when the fasting blood glucose value of more than 16.7 mmol/L was measured at 2 weeks after STZ injection.

### Isolation and Cultivation of EPCs

Bone marrow was harvested from 4-week-old male SD rats [Hubei Research Center of Laboratory Animals, SCXK [E] 2015-0018, Wuhan, China] after flushing with phosphate-buffered saline (PBS). The suspension was added into lymphoprep (Axis-Shield, Oslo, Norway) and centrifuged at 400 *g* for 30 min. Immed iately after isolation, mononuclear cells were washed twice with PBS and seeded on fibronectin (Sigma-Aldrich, St. Louis, MO)-coated 25-cm^2^ culture flasks (Corning Inc., NY) at a density of 2 × 10^7^ cells/flask, which cultured with EGM-2MV Bullet Kit medium (Lonza, MD, USA) at 37°C under 5% CO_2_. We changed the culture medium every 3 days. We identified EPCs by their property of uptaking acetylated LDL (ac-LDL) and Ulex europaeus agglutinin-1 (UEA-1). Cells with double positive staining were identified as differentiating EPCs (18).

### Lentivirus (LV) Transfection

The lentivirus vectors used in our study were procured from the commercial sources (Genechem Co., Ltd., Shanghai, China). Lentivirus constructs that express the GFP and APN gene to get the LV-GFP-APN vector, the vector without insertion of APN gene, was used as control (LV-GFP). Virus suspensions were stored at −80°C until transfection.

After being cultured for 7 days, EPCs were harvested and divided into three parts. One-third of the EPCs continued to be cultured (EPCs), one-third of the EPCs were transfected with LV-GFP-APN (LV-APN-EPCs), and one-third of the EPCs were transfected with LV-GFP (LV-EPCs). LV-APN-EPCs and LV-EPCs were seeded in 25-cm^2^ culture flasks (1 × 10^5^ cells/flask) and preincubated for 12 h with 4 ml of the EGM-2MV medium at 37°C under 5% CO_2_, respectively. After 12 h of culture, the medium was removed, and 2.5 ml of medium without FBS was replaced in the flasks. 5 × 10^8^ LV-GFP-APN with 20 μl and 5 μg/ml polybrene were added into the cells (LV-APN-EPCs) and 1 × 10^9^ LV-GFP with 10 μl and 5 μg/ml polybrene were added into the cells (LV-EPCs) and incubated for 12 h. Then, 2 ml of PBS was used to wash the cells twice to remove any free lentivirus, and 4 ml of fresh medium was added to the flasks, cultured at 37°C under 5% CO_2_. After 72 h of culture, we visualized enhanced green fluorescent protein (EGFP) by fluorescence microscopy to evaluate the results of the lentivirus transfection (18). We measured APN expression from EPCs after gene transduction. Expression of APN was detected by Western blot analysis (18).

### MCAO Models

Seventy T2DM rats were divided into five groups at random: (1) sham group (sham-operated rats, *n* = 14); (2) PBS group (T2DM rats treated with MCAO and PBS, *n* = 14); (3) LV-APN-EPCs group (T2DM rats treated with MCAO and LV-APN-EPCs, *n* = 14); (4) LV-EPCs group (T2DM rats treated with MCAO and LV-GFP-EPCs, *n* = 14); (5) EPCs group (T2DM rats treated with MCAO and EPCs, *n* = 14).

Fifty-six T2DM rats were subjected to transient (2 h) left MCAO *via* intraluminal vascular occlusion, as previously described (20). In brief, A nylon filament (heparin-dampened, 0.30 mm diameter, Beijing Cinontech Biotech Co., Ltd., Beijing, China) was put into the left common carotid artery (CCA) lumen and softly inserted into the internal carotid artery (ICA) for about 18 mm. Two hours later, reperfusion was initiated by thread withdrawal. Fourteen T2DM rats were subjected to sham operations; the procedure was the same as that of MCAO except for the insertion of a nylon filament. The exclusion criteria were MCAO rats on a five-point scale (“0”: no apparent deficits; “1”: left forelimb flexion; “2”: a decreased grip of the left forelimb while the tail was pulled; “3”: spontaneous movement in all directions; “4”: spontaneous left circling) (21). Rats who score <1 (possibly small to no lesion) or > 3 (poor survival) at 2 h after MCAO (before treatment) were excluded. At 1 h after reperfusion, LV-APN-EPCs, LV-EPCs, and EPCs resuspended in PBS were injected into the tail vein of rats (1 × 10^6^/ml per rat, LV-APN-EPCs group, LV-EPCs group and EPCs group), whereas 1 ml of PBS was IV-administered to the rats (PBS group and Sham group). T2-weighted imaging (T2WI) was performed to verify the ischemic stroke after treatment.

### Neurological Functional Scoring

The modified neurological severity score (mNSS) was evaluated before MCAO and on days 1, 7, and 14 after MCAO by an observer, who was blinded to the groups. Table 1 shows the mNSS points (22). Neurological function was graded as 0–18 (0, normal score; 18, most severe score).

**Table 1:** Modified neurological severity score points.

Motor tests	
Raising rat by tail	3
Flexion of forelimb	1
Flexion of hindlimb	1
Head moved > 10^°^ to vertical axis within 30 s	1
Placing rat on floor (normal = 0; maximum = 3)	3
Normal walk	0
Inability to walk straight	1
Circling toward paretic side	2
Falls down to paretic side	3
Sensory tests	2
Placing test (visual and tactile test)	1
Proprioceptive test (deep sensation, pushing paw against table edge to stimulate limb muscles)	1
Beam balance tests (normal = 0; maximum = 6)	6
Balances with steady posture	0
Grasps side of beam	1
Hugs beam and 1 limb falls down from beam	2
Hugs beam and 2 limbs fall down from beam, or spins on beam (>60 s)	3
Attempts to balance on beam but falls off (>40 s)	4
Attempts to balance on beam but falls off (>20 s)	5
Falls off; no attempt to balance or hang on to beam (<20 s)	6
Reflex absence and abnormal movements	4
Pinna reflex (head shake when auditory meatus is touched)	1
Corneal reflex (eye blink when cornea is lightly touched with cotton)	1
Startle reflex (motor response to a brief noise from snapping a clipboard paper)	1
Seizures, myoclonus, myodystony	1
Maximum points	18

*One point is awarded for inability to perform the tasks or for lack of a tested reflex: 13–18, severe injury; 7–12, moderate injury; 1–6, mild injury*.

### Magnetic Resonance Imaging (MRI) Measurements

All of the animals underwent brain MRI scans using a 7.0-T small-animal MR scanner (Bruker PharmaScan, Ettlingen, Germany) on day 14 post MCAO to measure the infarct rate. Brain scanning was conducted through T2WI sequence with the following parameters: a matrix of 256 × 256, echo time (TE) = 36 ms, repetition time (TR) = 3,000 ms, field of view (FOV) = 3 × 3 cm^2^, a total of 24 slices with a slice thickness of 0.8 mm, and the total sequence time was ~9 min. The infarct rate was determined on T2WI with the use of Image-Pro plus 6.0 software (Media Cybernetics Inc., Bethesda, MD, USA). The calculation of infarct rate was derived from the following formula (23): Infarct rate = (area of the control hemisphere—area of the non-infarcted region in the lesioned hemisphere)/area of the control hemisphere × 100%.

### Hematoxylin and Eosin (H and E) Staining

After brain scanning, the rats were anesthetized with 3% pentobarbital sodium. Five rats were randomly selected from each group and transcardially perfused with 4% paraformaldehyde solution and sacrificed using decapitation. The brains were immediately removed and post-fixed overnight in 4% paraformaldehyde and embedded in paraffin after full dehydration. Then, the brains were cut at 4-μm-thick coronal sections and the sections were dewaxed before use. H&E staining was done to view the pathological changes in the peri-infarct region of T2DM stroke rats. Abnormal neurons were shrunk and deeply stained, while normal neurons were orderly arranged with normal morphology and evident nucleus and nucleolus. We observed pathological changes by counting the number of viable normal neurons in the field.

### Terminal dUTP Nick-End Labeling (TUNEL) Staining

Cellular apoptosis was detected using immunofluorescent staining of TUNEL assays in the peri-infarct cortex. It was performed following the manufacturer's instructions (Yeasen, Shanghai, China). TUNEL-positive cells and DAPI-stained nuclei were counted by the Image-Pro Plus 6.0 software. We calculated the percentage of the positive cells using the following formula: Apoptosis rate = TUNEL-positive cells/total cells per field × 100%.

### Western Blotting Analysis

On day 14 post MCAO, five rats were randomly selected from each group and brains of the rats were taken after decollation. The cortex tissues of peri-infarct region were isolated on the ice rapidly and transferred to a refrigerator at −80°C for storage immediately. Total protein samples were extracted from the cortex of peri-infarct region, which was preserved in liquid nitrogen (−196°C). Protein concentrations were tested by using BCA Protein Assay Kit (Pierce, USA). The primary antibodies of anti-Bcl-2 (1:1,000, CST) and anti-Bax (1:1,000, Abcam) were used. GAPDH served as a loading control. In brief, 40-μg protein samples were segregated by sodium dodecyl sulfate–polyacrylamide gel electrophoresis (SDS-PAGE) and transferred to polyvinylidene difluoride (PVDF) membranes (Millipore, USA), which were blocked shakily with 5% non-fat milk for 2 h and incubated with the primary antibodies of anti-Bcl-2 and anti-Bax overnight at 4°C. After being incubated for a night, the membranes were washed by Tris-buffered saline and Tween-20 (TBST) five times and incubated with horseradish peroxidase-conjugated secondary antibodies (1:50,000, Boster, Wuhan, China) at 37°C for 2 h. Finally, an enhanced chemiluminescence detection kit (Pierce, USA) was used to develop, and BandScan (Glyko, ProZyme, USA) was used to analyze the bands.

### CD31+ Microvessel Counting

Immunofluorescent staining was carried out for CD31 (a marker of endothelial cells, Abcam, Cambridge, MA, USA) on day 14; the microvessel counts were counted in the cortex of peri-infarct region from three random slices of each rat brain and five rats from each group. We counted the number of the CD31-positive cells per field to evaluate microvessel density in the cortex of the peri-infarct region by fluorescence microscopy. Two investigators who were blinded to the groups carried out this quantification.

### Enzyme-Linked Immunosorbent Assay (ELISA)

The levels of eNOS and vascular endothelial growth factor (VEGF) in the ischemic brain were quantified with ELISA kits, according to the manufacturer's protocols (Nanjing, China).

### Statistical Analysis

All statistical analyses were performed by using GraphPad Prism (version 7.0; GraphPad Software, La Jolla, CA) and SPSS 22.0 software (IBM Corp, Armonk, NY, USA). The *t*-test and one-way analysis of variance (ANOVA) were used to test the intergroup differences in means of two and multiple groups for normally distributed variables, respectively. The Mann–Whitney *U* test and Kruskal–Wallis test were used to test the intergroup difference in means of two and multiple groups for non-normally distributed variables, respectively. Data were presented as mean ± standard deviation. A two-tailed *P* < 0.05 was considered statistically significant.

## Results

### Evaluation of T2DM Model and MCAO Model

**Figure 1:**
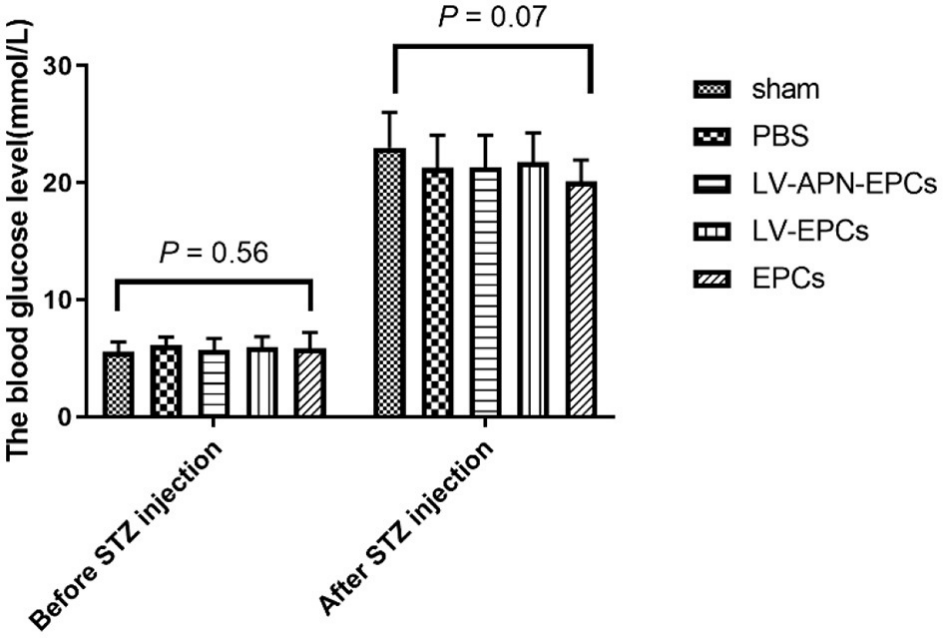
The fasting blood glucose in the rats of the five groups the day before STZ injection and 2 weeks after STZ injection (*n* = 14 per group). There was no significant intergroup difference both before (*P* = 0.56) and after STZ injection (*P* = 0.07). The fasting blood glucose in all rats was more than 16.7 mmol/L after STZ injection (*P* < 0.05 vs. before STZ injection).

The fasting blood glucose in the rats (*n* = 70) of the five groups was measured before STZ injection and 2 weeks after STZ injection. There was no significant intergroup difference both before (*P* = 0.56) and after STZ injection (*P* = 0.07). The fasting blood glucose in all rats was more than 16.7 mmol/L after STZ injection (*P* < 0.05 vs. before STZ injection) (Figure 1). After the establishment of the T2DM model, the rats appeared with polyuria, polydipsia, polyphagia, and weight loss as well as frequent fluctuations in high blood glucose.

The MCAO model was verified by brain scanning with T2WI sequence 24 h after transplantation. The success rate of this bolt line method was almost 100%, and the ischemic regions were nearly the same. In the process of surgery, seven rats died of vagus nerve damage and excessive bleeding. After MCAO, four rats were excluded from this study according to a five-point scale. During the following 14 days, nine rats died. Eventually, 50 rats were included in this study, with 10 rats in each group.

### Improved Functional Recovery of T2DM Stroke Rats After LV-APN-EPCs Treatment

**Figure 2:**
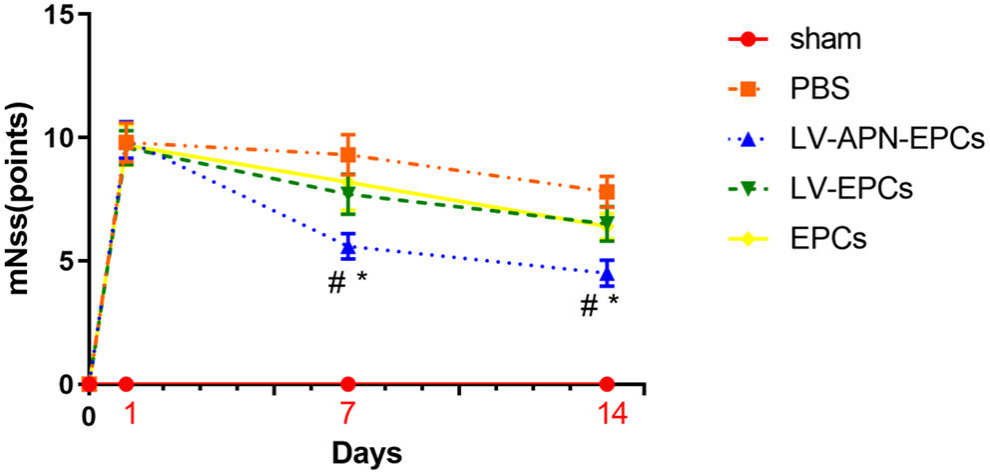
LV-APN-EPCs treatment significantly improves the neurological function of T2DM stroke rats. The mNSS before MCAO and on days 1, 7, and 14 after MCAO (*n* = 10 per group). #*P* < 0.05 vs. PBS group and *P < 0.05 vs. LV-EPCs and EPCs group.

On day 7 after the establishment of MCAO models, a significant reduction of neurological functional deficits was found in the LV-APN-EPCs treatment group, compared with the control or EPCs or LV-EPCs treatment groups [(5.60 ± 0.52) vs. (7.70 ± 0.82), (8.20 ± 1.14), (9.30 ± 0.82), *P* < 0.05]. On day 14, LV-APN-EPCs treatment significantly decreased neurological functional deficits, compared with the control or EPCs or LV-EPCs treatment groups [(4.50 ± 0.53) vs. (6.40 ± 0.52), (6.50 ± 0.71), (7.80 ± 0.63), *P* < 0.05] (Figure 2).

### Decreased Infarct Rate of T2DM Stroke Rats After LV-APN-EPCs Treatment

**Figure 3:**
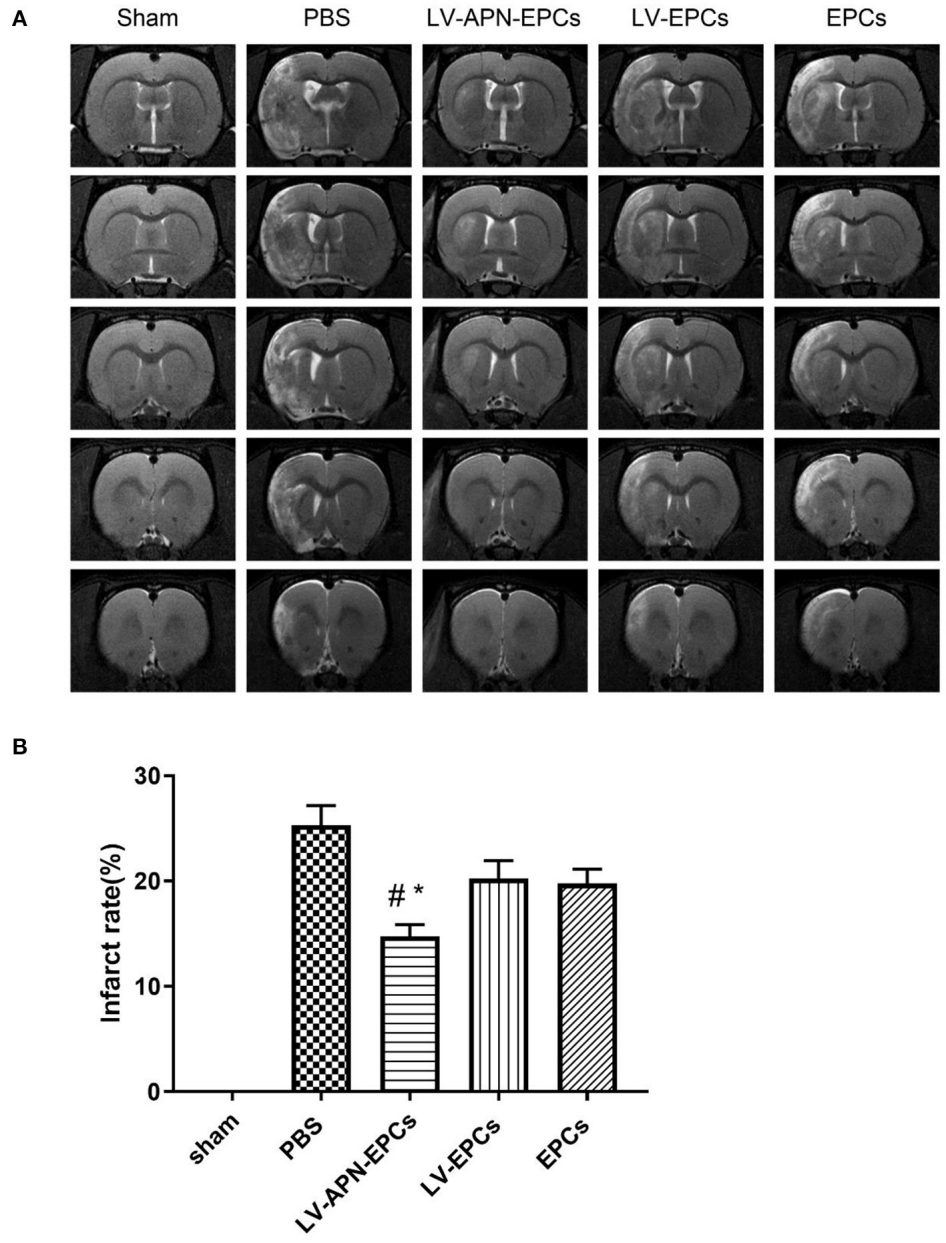
LV-APN-EPCs treatment significantly decreases infarct rate of T2DM stroke rats. **(A)** Representative T2WI of rats in various groups on day 14 post-stroke. **(B)** Quantitative analysis of infarct rate of animals (*n* = 10 per group). #*P* < 0.05 vs. PBS group and *P < 0.05 vs. LV-EPCs and EPCs group.

The infarct areas are shown as hyperintensity on T2WI (Figure 3A). LV-APN-EPCs treatment effectively decreased the infarct rate compared with the PBS treatment, LV-EPCs treatment, and EPCs treatment groups on day 14 [(14.74 ± 1.11) vs. (25.29 ± 1.88), (20.24 ± 1.71), (19.75 ± 1.37), *P* < 0.05] (Figure 3B).

### Ameliorated Morphological Damage of T2DM Stroke Rats After LV-APN-EPCs Treatment

**Figure 4:**
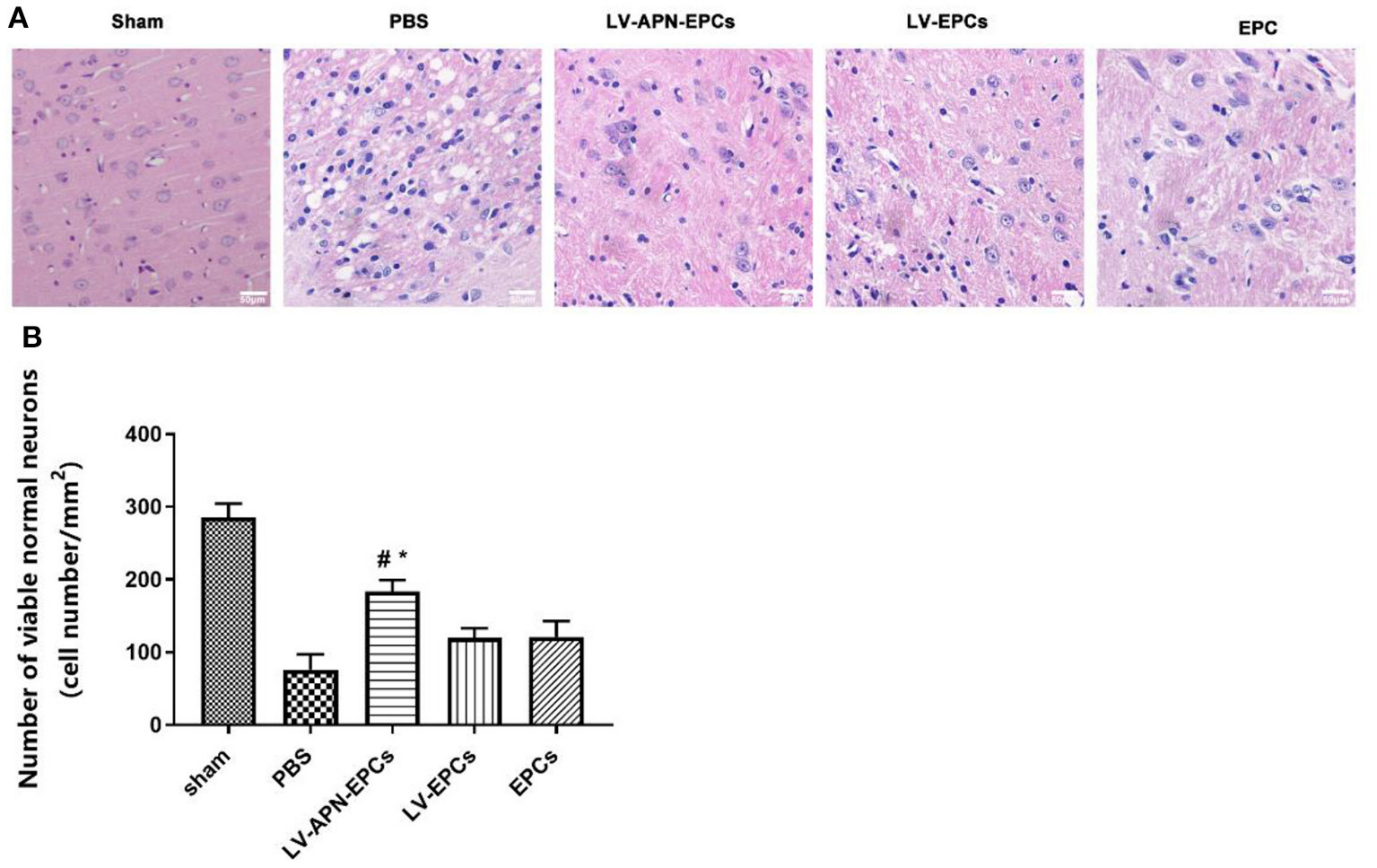
LV-APN-EPCs administration significantly ameliorates morphological damage in T2DM stroke rats on day 14. **(A)** Representative H&E staining of the peri-infarct region of T2DM stroke rats. magnification, 400×. **(B)** Quantitative analysis for the number of viable normal neurons of the different groups (*n* = 5 per group). #*P* < 0.05 vs. PBS group, *P < 0.05 vs. LV-EPCs and EPCs group.

The brains of T2DM Stroke rats after PBS treatment exhibited an edematous morphology with loose organization, decreased cell size, cell disorder, and nuclear pyknosis in H&E staining, and this condition was significantly alleviated by treatment with LV-APN-EPCs (Figure 4A). Abnormal neurons were shrunk and deeply stained, while normal neurons were orderly arranged with normal morphology and evident nucleus and nucleolus. LV-APN-EPCs treatment significantly increased the number of viable normal neurons compared with PBS treatment, LV-EPCs treatment, and EPCs treatment groups on day 14 [(183.60 ± 15.44)/mm^2^ vs. (76.00 ± 21.13)/mm^2^, (121.20 ± 21.58)/mm^2^, (120.00 ± 13.17)/mm^2^, *P* < 0.05] (Figure 4B).

### Decreased Cellular Apoptosis of T2DM Stroke Rat After LV-APN-EPCs Treatment

**Figure 5:**
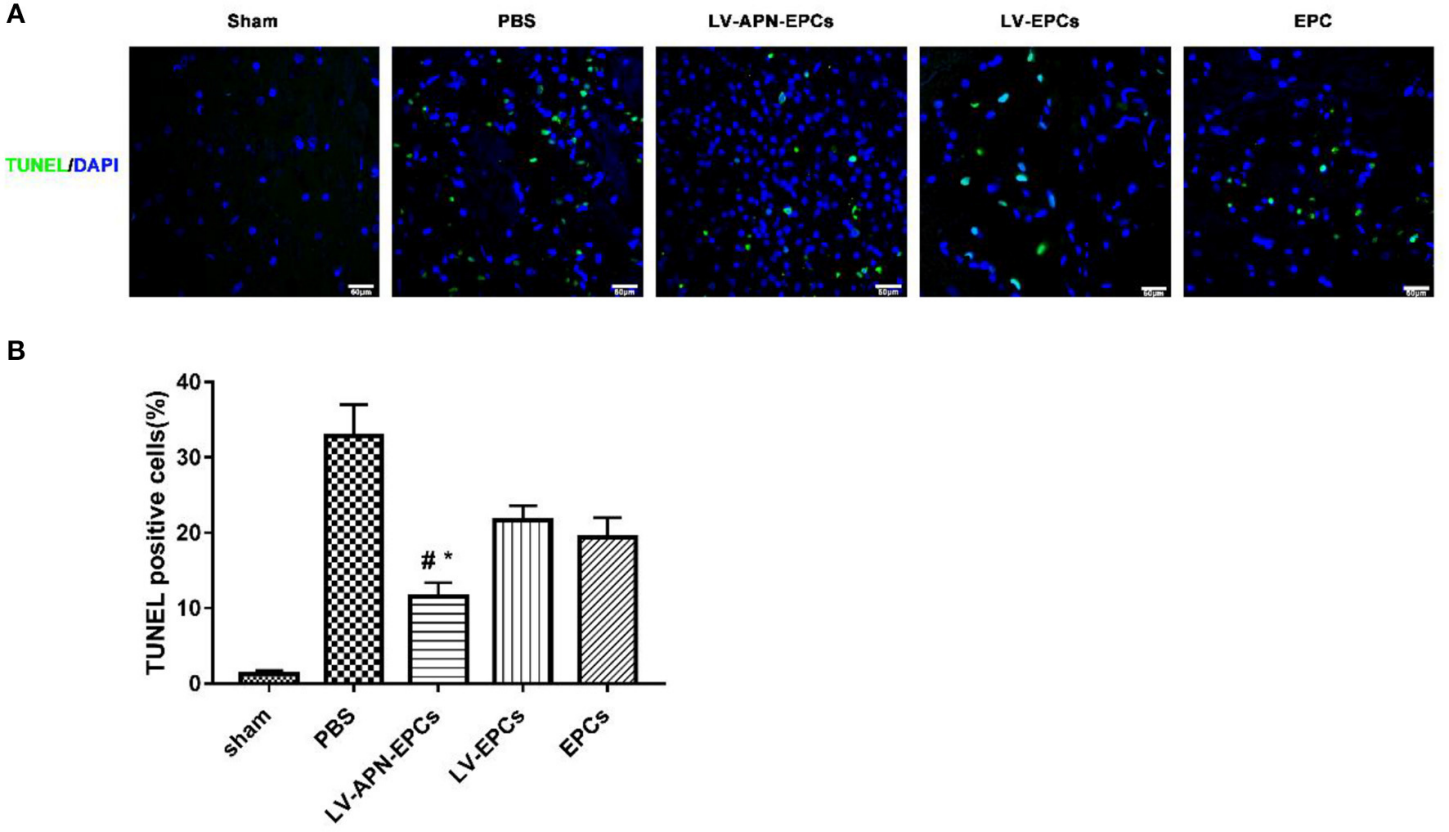
LV-APN-EPCs administration significantly decreases neuronal apoptosis in T2DM stroke rats on day 14. **(A)** Representative TUNEL staining of the peri-infarct region of T2DM stroke rats. Magnification, 400×. **(B)** Quantitative analysis for the apoptotic ratio of TUNEL-positive cells of the different groups (*n* = 5 per group). #*P* < 0.05 vs. PBS group, *P < 0.05 vs. LV-EPCs and EPCs group.

According to the results of TUNEL labeling on day 14 after MCAO, LV-APN-EPCs treatment significantly decreased the amount of TUNEL-positive cells, compared with control or EPCs or LV-EPCs treatment [(11.57 ± 1.82)% vs. (32.89 ± 4.13)%, (19.46 ± 2.56)%, (21.65 ± 1.97)%, *P* < 0.05] (Figure 5).

**Figure 6:**
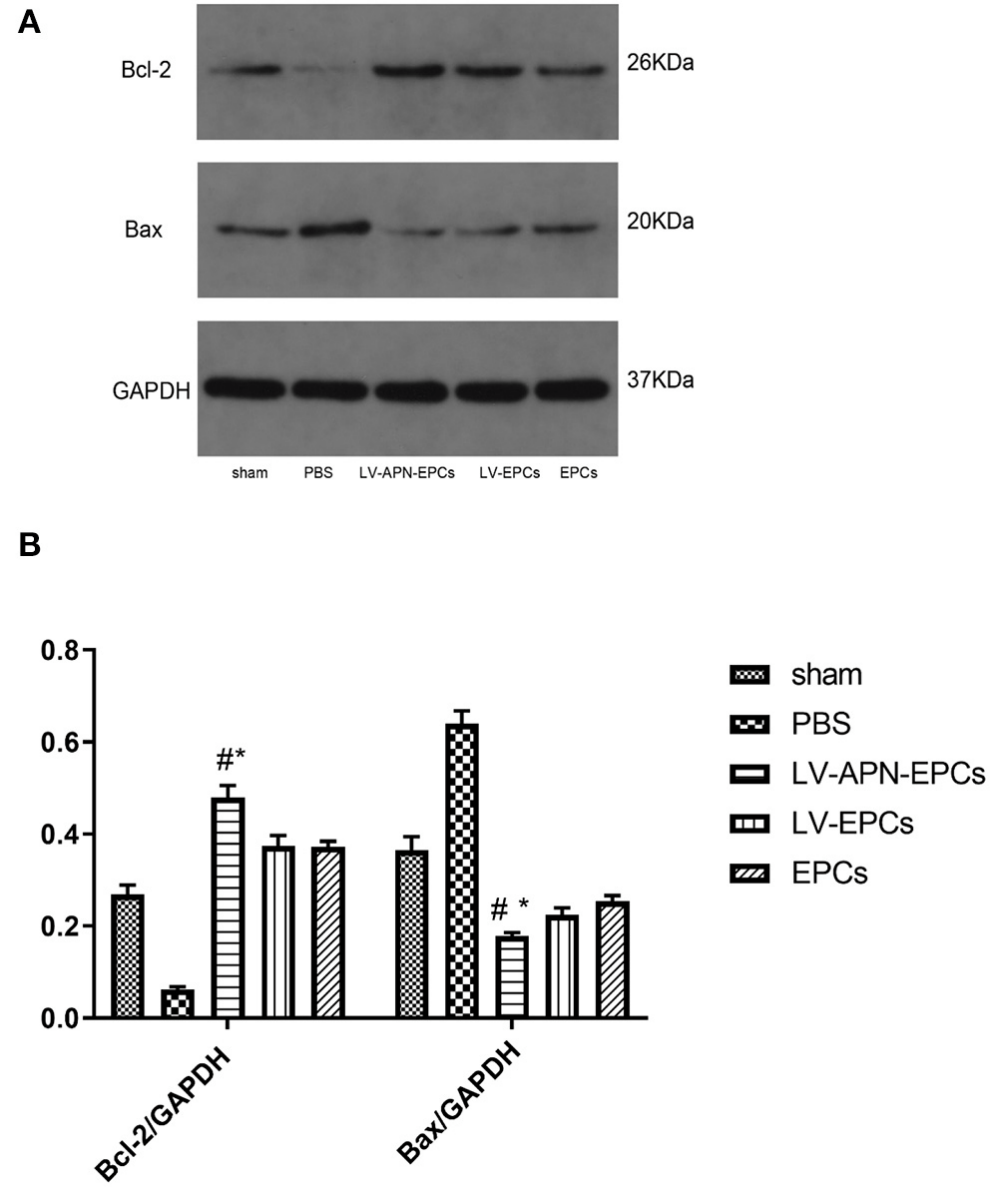
LV-APN-EPCs treatment regulates apoptosis-related proteins expression in the peri-infarct cortex of T2DM stroke rats on day 14. **(A)** Levels of Bcl-2 and Bax detected by Western blotting. **(B)** Quantification of Bcl-2 and Bax expression in the ischemic brain (*n* = 5 per group). #*P* < 0.05 vs. PBS group, *P < 0.05 vs. LV-EPCs and EPCs group.

The expressions of antiapoptotic protein Bcl-2 and proapoptotic protein Bax were tested in the peri-infarct cortex on day 14 after stroke to further research how LV-APN-EPCs treatment suppressed cellular apoptosis. Western blotting results showed that LV-APN-EPCs treatment significantly increased the expression of Bcl-2 protein compared with the PBS group [(0.48 ± 0.03) vs. (0.06 ± 0.01), *P* < 0.05] and reduced the expression of Bax when compared with the PBS group [(0.18 ± 0.01) vs. (0.64 ± 0.03), *P* < 0.05] (Figure 6). These results indicated that LV-APN-EPCs treatment may inhibit cellular apoptosis through regulating the expression of apoptosis-related proteins in the peri-infarct cortex.

### Increased Angiogenesis of T2DM Rat After LV-APN-EPCs Treatment

**Figure 7:**
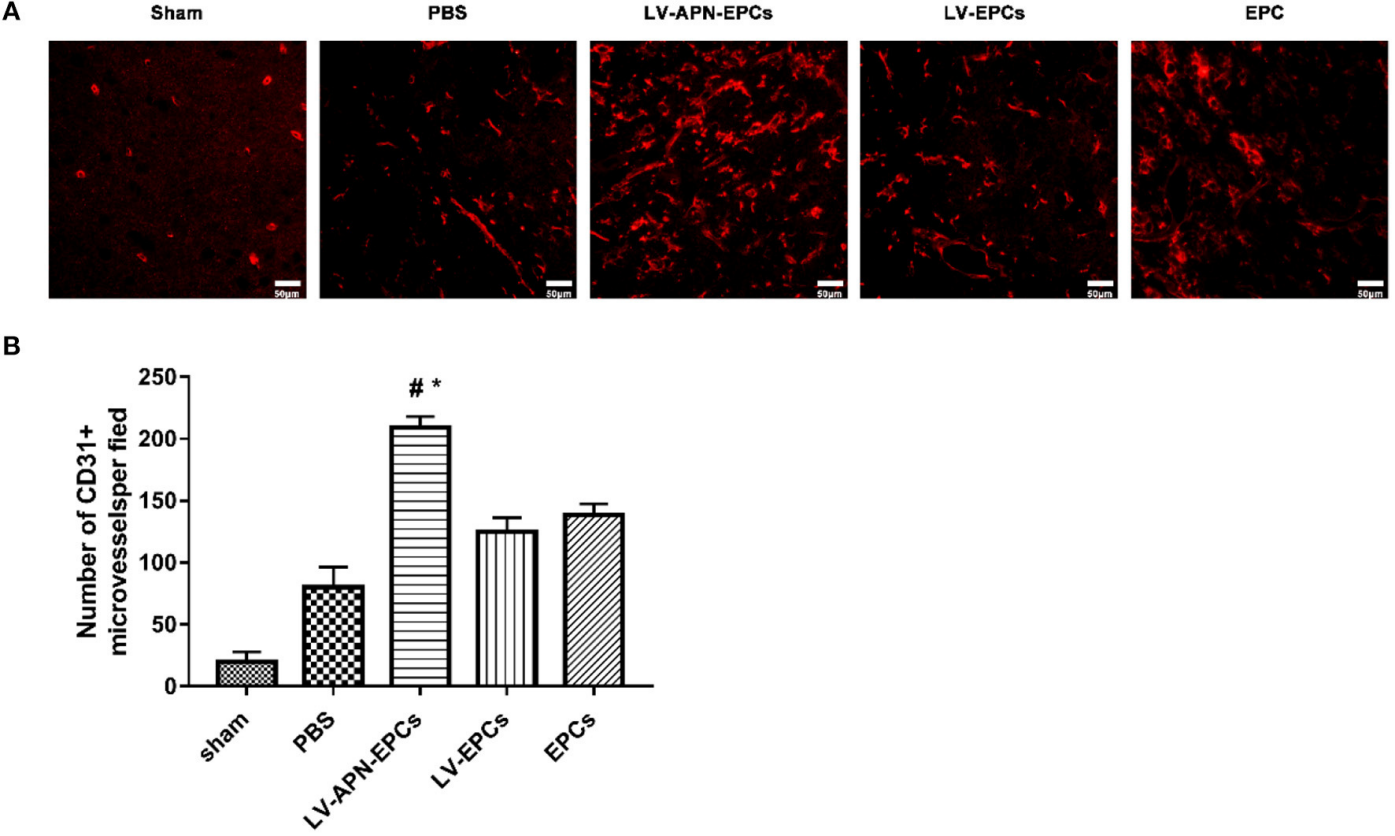
LV-APN-EPCs administration significantly increases angiogenesis in T2DM stroke rats on day 14. **(A)** Representative CD31 immunofluorescent staining of the peri-infarct region of T2DM stroke rats. Magnification, 400×. **(B)** Quantitative data for the microvessel counts of rats brain (*n* = 5 per group). #*P* < 0.05 vs. PBS group, *P < 0.05 vs. LV-EPCs and EPCs group.

Compared to the PBS or EPCs or LV-EPCs treatment groups, a higher level of microvessel density in the peri-infarct region of the cortex was observed in the LV-APN-EPCs treatment group on day 14 after MCAO establishment [(80.20 ± 16.22), (138.40 ± 9.15), (125.00 ± 11.29) vs. (209.00 ± 9.25), *P* < 0.05] (Figure 7).

### Altered Expression of Cytokines in T2DM Rat After LV-APN-EPCs Treatment

**Figure 8:**
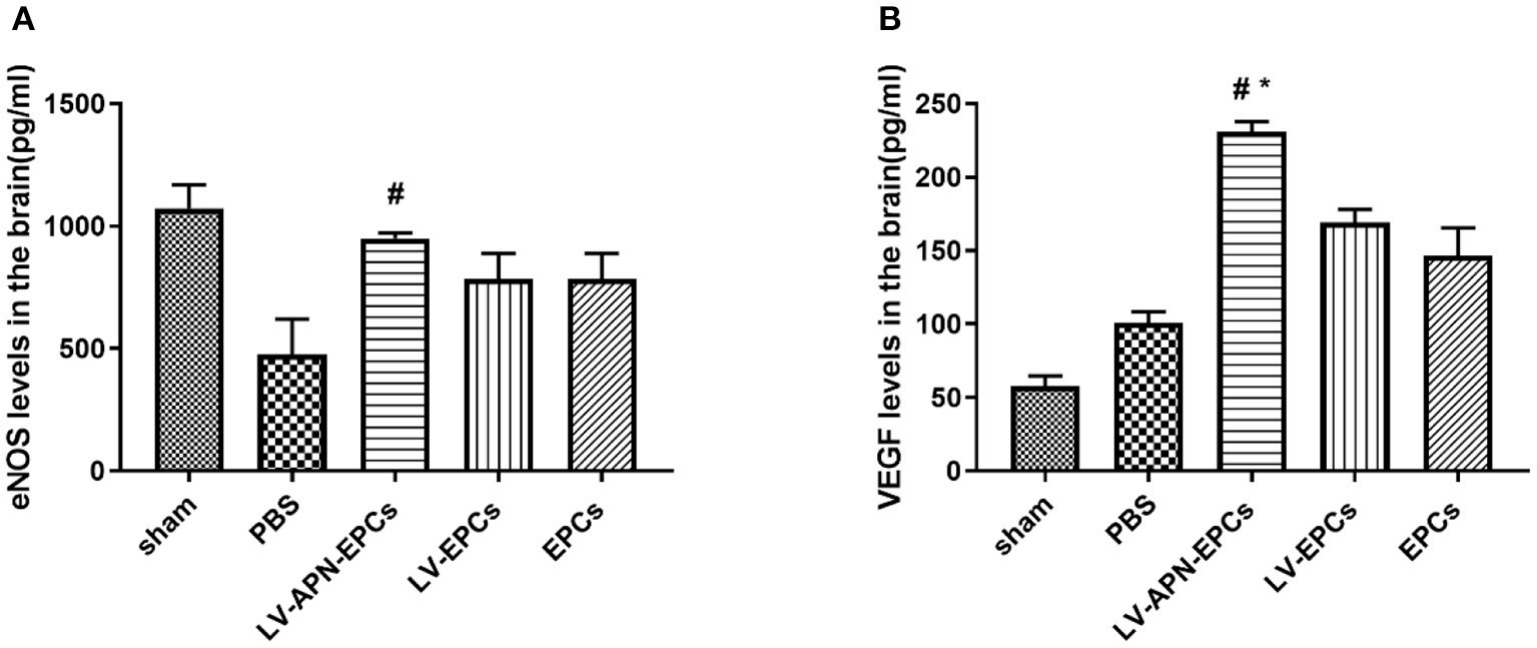
The expression of eNOS **(A)** and VEGF **(B)** was detected by ELISA in T2DM stroke rats (*n* = 5 per group). #*P* < 0.05 vs. PBS group, *P < 0.05 vs. LV-EPCs and EPCs group.

Compared with the PBS or EPCs or LV-EPCs treatment groups, significantly higher levels of eNOS expression based on ELISA assessment were detected in the LV-APN-EPCs treatment group on day 14 after MCAO establishment [(475.13 ± 145.69), (785.71 ± 103.07), (786.14 ± 103.84) vs. (951.25 ± 20.99) pg/ml, *P* < 0.05] (Figure 8A) and similarly higher levels of VEGF expression were detected in the LV-APN-EPCs treatment group [(101.15 ± 7.19), (169.00 ± 9.33), (146.65 ± 18.78) vs. (231.03 ± 6.81) pg/ml, *P* < 0.05] (Figure 8B).

## Discussion

In the current study, the T2DM rat model was induced by a high-fat diet and a low dose of STZ, which cause blood glucose in a high condition (24, 25). The induction of stroke model was achieved using the bolt line method, and the withdrawal of the filament was performed to reach reperfusion 2 h after MCAO (26); this process resembles what occurs during ischemia–reperfusion injury in clinical scenes. Above all, it is beneficial for the assessment of therapeutic efficacy because of the equal infarct rate induced by the method. MRI has been used to non-invasively evaluate the ischemic stroke in animals (27) and patients (28). In the present study, we used T2WI to non-invasively assess the infarct rate. Our study showed that LV-APN-EPCs treatment effectively decreased the infarct rate 2 weeks after stroke in T2DM stroke rats.

Decreased cellular apoptosis was found in the LV-APN-EPCs treatment group, compared with PBS or EPCs or LV-EPCs treatment. APN attenuates high glucose-induced apoptosis (29), which is consistent with our findings. Our data indicated that LV-APN-EPC treatment reduced cellular apoptosis, which significantly decreased the amount of TUNEL-positive cells. As apoptosis plays a vital part in the death of cell after stroke, especially in the peri-infarct region (30), inhibiting apoptosis might be an effective approach to improve the survival rate of cells and finally to contribute to the recovery after MCAO. Stem cell transplantation can suppress cellular apoptosis of stroke animals in previous studies (31). Apoptosis was inhibited by downregulating expression of Bax and upregulating expression of Bcl-2 (32). In the current study, we found that LV-APN-EPCs treatment increased the level of Bcl-2 protein and reduced the expression of Bax.

In this study, compared with PBS or EPCs or LV-EPCs treatment, neurological functional deficits were reduced and morphological damage was ameliorated in the LV-APN-EPCs treatment group. According to the results of the CD31 immunofluorescent staining of the peri-infarct region of T2DM stroke rats, a higher level of microvessel density was observed in the LV-APN-EPCs treatment group, which suggested that increased angiogenesis may play an important mechanistic role in protecting against ischemic stroke.

Ischemic stroke decreases cerebral blood flow and triggers vascular remodeling, thus improving blood supply by angiogenesis (33). However, diabetes mellitus leads to vascular endothelial cell dysfunction and exuberant angiogenesis, but it is also conducive to dysfunctional neovascularization and poor recovery in ischemic stroke. EPCs can contribute to vascular endothelial repair and angiogenesis (34); increasing angiogenesis is associated with neurological functional outcome after stroke (35). Our *in vivo* study indicated that LV-APN-EPCs treatment had effects on the recovery of neurological functional deficits in diabetic stroke rats, but EPC transplantation alone had little effect. These results may have been interpreted that only a small amount of EPCs survived and homed to the ischemic region in the diabetic animals with the EPCs transplantation alone, as was proved in a previous study (36). In our previous study, we have reported that LV-APN-EPCs treatment may be a useful approach for ischemic stroke in non-diabetic rats (18). Nakamura et al. (37) found that adiponectin can promote the migration activity of EPCs, mainly through PI 3-kinase/Cdc42/Rac1. However, diabetes mellitus reduces the number of circulating EPCs (38), and a previous research has indicated that APN can counterbalance the phenomenon (17). On the other hand, EPCs from T2DM demonstrate impaired function including adhesion, proliferation, and formation of vessel structures (12). A recent study has revealed that APN has a vascular protective effect by improving the function of high glucose-suppressed EPCs in diabetic patients, and this effect is mediated partly by activating eNOS to increase NO production in the endothelial cells (39), and Huang et al. (40) also found that globular adiponectin could ameliorate high glucose-impaired EPC function in vasculogenesis by restoring eNOS activity and improved high glucose-impaired EPC function by NO and p38 MAPK-related mechanisms, which are consistent with our finding that LV-APN-EPCs treatment significantly increased the expression level of eNOS in diabetic rats.

In addition, transplanted EPCs can secrete proangiogenic factors, such as VEGF (41); the increased levels of VEGF may contribute to the enhanced angiogenesis after diabetic stroke. Previous studies have demonstrated that VEGF therapy from day 1 to 3 exacerbates ischemic injury due to the increased blood–brain barrier leakage. However, from day 7 to 21 of cerebral ischemia, increased VEGF is beneficial; it generates neovessels and accelerates their maturation and stabilization (42). In the present study, the increased VEGF may be exerted mainly by EPCs and may play an effective role.

## Conclusions

In conclusion, we investigated the effects of intravenously transplanted LV-GFP-APN prelabeled EPCs into T2DM rats initiated 1 h after ischemia–reperfusion injury. We demonstrated that the combination of EPCs transplantation and APN gene synergistically improved neurological functional deficits, reduced infarct rate, alleviated morphological damage, and decreased neuronal apoptosis, which had neuroprotective effects on T2DM stroke rats. Our data suggest that the underlying mechanism of LV-APN-EPCs induced benefits that may promote the angiogenesis effect.

## Data Availability Statement

The data used to support the findings of this study are included within the article, further inquiries can be directed to the corresponding authors.

## Ethics Statement

All experimental procedures followed the Guide for the Care and Use of Laboratory Animals published by National Academies Press (US) (8th edition, 2011). The use of animals was approved by the Animal Ethics Committee of the Medical School of Wuhan University.

## Author Contributions

MW: conceptualization, data curation, and writing—original draft. YL, RZ, SZ, and HF: methodology. HF: statistical analyses. ZK: visualization. NA: validation. ZL: formal analysis. QC: resources. YH and YL: supervision and writing—review and editing. All authors contributed to the article and approved the submitted version.

## Conflict of Interest

The authors declare that the research was conducted in the absence of any commercial or financial relationships that could be construed as a potential conflict of interest.

## References

[1] Virani SS Alonso A Benjamin EJ Bittencourt MS Callaway CW Carson AP. Heart disease and stroke statistics-2020 update: a report from the American Heart Association. Circulation. (2020) 141:e139–596. 10.1161/CIR.0000000000000757 31992061

[2] O'Donnell MJ Chin SL Rangarajan S Xavier D Liu L Zhang H. Global and regional effects of potentially modifiable risk factors associated with acute stroke in 32 countries (INTERSTROKE): a case-control study. Lancet. (2016) 388:761–75. 10.1016/S0140-6736(16)30506-2 27431356

[3] Emerging Risk Factors Collaboration Sarwar N Gao P Seshasai SR Gobin R Kaptoge S. Diabetes mellitus, fasting blood glucose concentration, and risk of vascular disease: a collaborative meta-analysis of 102 prospective studies. Lancet. (2010) 375:2215–22. 10.1016/S0140-6736(10)60484-9 20609967 PMC2904878

[4] Zhang L Chopp M Zhang Y Xiong Y Li C Sadry N. Diabetes mellitus impairs cognitive function in middle-aged rats and neurological recovery in middle-aged rats after stroke. Stroke. (2016) 47:2112–8. 47:2112–8. 10.1161/STROKEAHA.115.012578 27387991 PMC4961558

[5] Zhu S McClure LA Lau H Romero JR White CL Babikian V. Recurrent vascular events in lacunar stroke patients with metabolic syndrome and/or diabetes. Neurology. (2015) 85:935–41. 85:935–41. 10.1212/WNL.0000000000001933 26296518 PMC4567462

[6] De Silva DA Ebinger M Christensen S Parsons MW Levi C Butcher K. Baseline diabetic status and admission blood glucose were poor prognostic factors in the EPITHET trial. Cerebrovasc Dis. (2010) 29:14–21. 29:14–21. 10.1159/000255969 19893307

[7] Huang L Liu Y Lu J Cerqueira B Misra V Duong TQ. Intraarterial transplantation of human umbilical cord blood mononuclear cells in hyperacute stroke improves vascular function. Stem Cell Res Ther. (2017) 8:74. 8:74. 10.1186/s13287-017-0529-y 28330501 PMC5361847

[8] Savitz SI. Developing cellular therapies for stroke. Stroke. (2015) 46:2026–31. 46:2026–31. 10.1161/STROKEAHA.115.007149 26045599 PMC4479962

[9] Asahara T Murohara T Sullivan A Silver M van der Zee R Li T. Isolation of putative progenitor endothelial cells for angiogenesis. Science. (1997) 275:964–7. 275:964–7. 10.1126/science.275.5302.964 9020076

[10] Ma Y Jiang L Wang L Li Y Liu Y Lu W. Endothelial progenitor cell transplantation alleviated ischemic brain injury *via* inhibiting C3/C3aR pathway in mice. J Cereb Blood Flow Metab. (2019) 40:2374–86. 40:2374–86. 10.1177/0271678X19892777 31865842 PMC7820683

[11] Fadini GP Mehta A Dhindsa DS Bonora BM Sreejit G Nagareddy P. Circulating stem cells and cardiovascular outcomes: from basic science to the clinic. Eur Heart J. (2019) 41:4271–82. 41:4271–82. 10.1093/eurheartj/ehz923 31891403 PMC7825095

[12] Tepper OM Galiano RD Capla JM Kalka C Gagne PJ Jacobowitz GR. Human endothelial progenitor cells from type II diabetics exhibit impaired proliferation, adhesion, and incorporation into vascular structures. Circulation. (2002) 106:2781–6. 106:2781–6. 10.1161/01.CIR.0000039526.42991.93 12451003

[13] Benomar Y Amine H Crepin D Al Rifai S Riffault L Gertler A. Central Resistin/TLR4 impairs adiponectin signaling, contributing to insulin and FGF21 resistance. Diabetes. (2016) 65:913–26. 65:913–26. 10.2337/db15-1029 26740596

[14] Nishimura M Izumiya Y Higuchi A Shibata R Qiu J Kudo C. Adiponectin prevents cerebral ischemic injury through endothelial nitric oxide synthase dependent mechanisms. Circulation. (2008) 117:216–23. 117:216–23. 10.1161/CIRCULATIONAHA.107.725044 18158361

[15] Cheng KK Lam KS Wang Y Huang Y Carling D Wu D. Erratum. Adiponectin-induced endothelial nitric oxide synthase activation and nitric oxide production are mediated by APPL1 in endothelial cells. Diabetes. (2007) 56:1387–94. 10.2337/db06-1580 17287464

[16] Hong Y Yu Q Kong Z Wang M Zhang R Li Y. Exogenous endothelial progenitor cells reached the deficient region of acute cerebral ischemia rats to improve functional recovery *via* Bcl-2. Cardiovasc Diagn Ther. (2020) 10:695–704. 10.21037/cdt-20-329 32968626 PMC7487376

[17] Chang J Li Y Huang Y Lam KS Hoo RL Wong WT. Adiponectin prevents diabetic premature senescence of endothelial progenitor cells and promotes endothelial repair by suppressing the p38 MAP kinase/p16INK4A signaling pathway. Diabetes. (2010) 59:2949–59. 10.2337/db10-0582 20802255 PMC2963556

[18] Zhang R Xie X Yu Q Feng H Wang M Li Y. Constitutive expression of adiponectin in endothelial progenitor cells protects a rat model of cerebral ischemia. Neural Plast. (2017) 2017:6809745. 2017:6809745. 10.1155/2017/6809745 29201467 PMC5671740

[19] Alves BEO de Alencar AKN Gamba LER Trachez MM da Silva JS Araújo JSC. Reduction of cardiac and renal dysfunction by new inhibitor of DPP4 in diabetic rats. Pharmacol Rep. (2019) 71:1190–200. 71:1190–200. 10.1016/j.pharep.2019.07.005 31669883

[20] Longa EZ Weinstein PR Carlson S Cummins R. Reversible middle cerebral artery occlusion without craniectomy in rats. Stroke. (1989) 20:84–91. 10.1161/01.STR.20.1.84 2643202

[21] Shao M Shen Y Sun H Meng D Huo W Qi X. Protectiveness of artesunate given prior ischemic cerebral infarction is mediated by increased autophagy. Front Neurol. (2018) 9:634. 9:634. 10.3389/fneur.2018.00634 30174640 PMC6107698

[22] Chen J Sanberg PR Li Y Wang L Lu M Willing AE. Intravenous administration of human umbilical cord blood reduces behavioral deficits after stroke in rats. Stroke. (2001) 32:2682–8. 32:2682–8. 10.1161/hs1101.098367 11692034

[23] Swanson RA Morton MT Tsao-Wu G Savalos RA Davidson C Sharp FR. A semiautomated method for measuring brain infarct volume. J Cereb Blood Flow Metab. (1990) 10:290–3. 10.1038/jcbfm.1990.47 1689322

[24] Yan T Venkat P Chopp M Zacharek A Ning R Roberts C. Neurorestorative responses to delayed human mesenchymal stromal cells treatment of stroke in type 2 diabetic rats. Stroke. (2016) 47:2850–8. 47:2850–8. 10.1161/STROKEAHA.116.014686 27729575 PMC5134897

[25] Qiu ZK He JL Liu X Zhang GH Zeng J Nie H. The antidepressant-like activity of AC-5216, a ligand for 18KDa translocator protein (TSPO), in an animal model of diabetes mellitus. Sci Rep. (2016) 6:37345. 6:37345. 10.1038/srep37345 27886206 PMC5122851

[26] An H Duan Y Wu D Yip J Elmadhoun O Wright JC. Phenothiazines enhance mild hypothermia-induced neuroprotection *via* PI3K/Akt regulation in experimental stroke. Sci Rep. (2017) 7:7469. 7:7469. 10.1038/s41598-017-06752-5 28785051 PMC5547051

[27] Vieites-Prado A Iglesias-Rey R Fernandez-Susavila H da Silva-Candal A Rodriguez-Castro E Grohn OH. Protective effects and magnetic resonance imaging temperature mapping of systemic and focal hypothermia in cerebral ischemia. Stroke. (2016) 47:2386–96. 10.1161/STROKEAHA.116.014067 27491739

[28] Menjot de Champfleur N Saver JL Goyal M Jahan R Diener HC Bonafe A. Efficacy of stent-retriever thrombectomy in magnetic resonance imaging vs. computed tomographic perfusion-selected patients in SWIFT PRIME Trial (Solitaire FR with the intention for thrombectomy as primary endovascular treatment for acute ischemic stroke). Stroke. (2017) 48:1560–6. 10.1161/STROKEAHA.117.016669 28465460

[29] Wang Y Zhang J Zhang L Gao P Wu X. Adiponectin attenuates high glucose-induced apoptosis through the AMPK/p38 MAPK signaling pathway in NRK-52E cells. PLoS ONE. (2017) 12:e0178215. 12:e0178215. 10.1371/journal.pone.0178215 28542560 PMC5444659

[30] Sun Y Zhao D Yang Y Gao C Zhang X Ma Z. Adiponectin exerts cardioprotection against ischemia/reperfusion injury partially *via* calreticulin mediated anti-apoptotic and anti-oxidative actions. Apoptosis. (2017) 22:108–17. 10.1007/s10495-016-1304-8 27757734

[31] Bao C Wang Y Min H Zhang M Du X Han R. Combination of ginsenoside Rg1 and bone marrow mesenchymal stem cell transplantation in the treatment of cerebral ischemia reperfusion injury in rats. Cell Physiol Biochem. (2015) 37:901–10. 10.1159/000430217 26384017

[32] Seo TB Kim TW Shin MS Ji ES Cho HS Lee JM. Aerobic exercise alleviates ischemia-induced memory impairment by enhancing cell proliferation and suppressing neuronal apoptosis in hippocampus. Int Neurourol J. (2014) 18:187–97. 10.5213/inj.2014.18.4.187 25562035 PMC4280438

[33] Liu J Wang Y Akamatsu Y Lee CC Stetler RA Lawton MT. Vascular remodeling after ischemic stroke: mechanisms and therapeutic potentials. Prog Neurobiol. (2014) 115:138–56. 115:138–56. 10.1016/j.pneurobio.2013.11.004 24291532 PMC4295834

[34] Asahara T Masuda H Takahashi T Kalka C Pastore C Silver M. Bone marrow origin of endothelial progenitor cells responsible for postnatal vasculogenesis in physiological and pathological neovascularization. Circ Res. (1999) 85:221–8. 10.1161/01.RES.85.3.221 10436164

[35] Ueno Y Hira K Miyamoto N Kijima C Inaba T Hattori N. Pleiotropic effects of exosomes as a therapy for stroke recovery. Int J Mol Sci. (2020) 21:6894. 10.3390/ijms21186894 32962207 PMC7555640

[36] Bai YY Wang L Peng XG Wang YC Chang D Zheng S. Non-invasive monitoring of transplanted endothelial progenitor cells in diabetic ischemic stroke models. Biomaterials. (2015) 40:43–50. 40:43–50. 10.1016/j.biomaterials.2014.11.018 25433605

[37] Nakamura N Naruse K Matsuki T Hamada Y Nakashima E Kamiya H. Adiponectin promotes migration activities of endothelial progenitor cells *via* Cdc42/Rac1. FEBS Lett. (2009) 583:2457–63. 583:2457–63. 10.1016/j.febslet.2009.07.011 19596003

[38] Chen J Cui X Zacharek A Cui Y Roberts C Chopp M. White matter damage and the effect of matrix metalloproteinases in type 2 diabetic mice after stroke. Stroke. (2011) 42:445–52. 42:445–52. 10.1161/STROKEAHA.110.596486 21193743 PMC3108495

[39] Chen H Montagnani M Funahashi T Shimomura I Quon MJ. Adiponectin stimulates production of nitric oxide in vascular endothelial cells. J Biol Chem. (2003) 278:45021–6. 278:45021–6. 10.1074/jbc.M307878200 12944390

[40] Huang PH Chen JS Tsai HY Chen YH Lin FY Leu HB. Globular adiponectin improves high glucose-suppressed endothelial progenitor cell function through endothelial nitric oxide synthase dependent mechanisms. J Mol Cell Cardiol. (2011) 51:109–19. 10.1016/j.yjmcc.2011.03.008 21439968

[41] He XY Chen ZZ Cai YQ Xu G Shang JH Kou SB. Expression of cytokines in rat brain with focal cerebral ischemia after grafting with bone marrow stromal cells and endothelial progenitor cells. Cytotherapy. (2011) 13:46–53. 10.3109/14653249.2010.510505 20735164

[42] Moisan A Favre IM Rome C Grillon E Naegele B Barbieux M. Microvascular plasticity after experimental stroke: a molecular and MRI study. Cerebrovasc Dis. (2014) 38:344–53. 38:344–53. 10.1159/000368597 25427570

